# Collective Dynamics of Frustrated Biological Neuron Networks

**DOI:** 10.1103/1258-cl48

**Published:** 2025-07-02

**Authors:** Guanyu Li, Ryan LeFebre, Alia Starman, Patrick Chappell, Andrew Mugler, Bo Sun

**Affiliations:** 1 Department of Physics, Oregon State University, Corvallis, Oregon 97331, USA; 2 Department of Physics and Astronomy, University of Pittsburgh, Pittsburgh, Pennsylvania 15260, USA; 3 Department of Biomedical Sciences, Carlson College of Veterinary Medicine, Oregon State University, Corvallis, Oregon 97331, USA

## Abstract

To maintain normal functionality, it is necessary for a multicellular organism to generate robust responses to external temporal signals. However, the underlying mechanisms to coordinate the collective dynamics of cells remain poorly understood. Here, we study the calcium activity of biological neuron networks excited by periodic ATP stimuli. We use micropatterning to control the cells’ physical connectivity. We find that whereas isolated cells become more synchronized in their calcium activity at long driving periods, connected cells become less synchronized, despite expressing more gap junctions which enable calcium exchange. To understand this result, we use a mathematical model in which a bifurcation analysis has previously shown coupling-induced desynchronization in an oscillatory network. Using parameters close to this bifurcation but in the excitable regime, we find that this desynchronization persists and can explain the experimental observations. The model further predicts that co-culturing with gap-junction-deficient cells should restore synchronization, which experiments confirm. Combining quantitative experiments, the physical and biological manipulation of cells, and mathematical modeling, our results suggest that cell-to-cell connectivity significantly affects how populations encode an external temporal signal as it slows down: Sparse networks synchronize due to longer entrainment, whereas highly connected networks can desynchronize due to dynamic frustration.

## I. INTRODUCTION

As a key task to sustain normal functionality, communities of living systems and multicellular organisms must produce coordinated responses to temporal signals from the microenvironment [1,2]. The period of the environmental stimuli may vary from seconds, such as in heartbeats [3], to hours, such as in mammalian circadian rhythm [4]. Unraveling the mechanisms that coordinate the individual responses to generate robust collective dynamics holds the key to understanding organizational principles in biology [5,6].

Synchronization is a prevalent form of the collective sensory response to temporal signals, and is observed in a vast array of living systems such as bacterial colonies [7], social amoebae [8], mammalian muscles [9], and the human brain [10]. For a multicellular system to synchronize, it relies on robust intercellular communication that couples cells through mechanical [11], chemical [12], or electrical means [13] in order to balance against intrinsic and extrinsic noise [14]. However, uncontrolled synchronization is not always desirable and can sometimes be fatal [15]. The fundamental question of how the same type of intercellular coupling can both promote and limit synchronization remains poorly understood.

In our previous studies, we found that various cell types, including fibroblasts [16], endothelial cells [17], and neural cells [18], all exhibit collective sensory responses to temporal signals, orchestrated through gap junctional communications among individual cells. In particular, the communication strength between cells is modulated by the period of external stimuli, and the information transfer between cells is minimized at complete synchronization [17,18]. Thus, gap junctions serve as dynamic and adaptable communication channels. When a multicellular organism depends on gap junctions to integrate the nonlinear dynamics of individual cells, as in the case of the biological neural network we will study here, it gives rise to complex network dynamics that remain incompletely characterized [19,20].

In this article we combine quantitative experiments and computational modeling to investigate the synchronization of communicating neuronal cells driven by periodic extracellular adenosine triphosphate (ATP) stimuli. We examine the calcium dynamics of KTaR cells, a neuronal cell line we derived from KNDy (kisspeptin, neurokinin B, and dynorphin) neurons within the arcuate nucleus of an adult female mouse [21]. ATP-induced kisspeptin neuron dynamics regulate a variety of mammalian reproduction functions such as luteinizing hormone surge and ovulation [22]. Here, we control the communication between KTaR cells physically through cell-adhesive micropatterning, and biologically through CRISPR-Cas9 knockout of gap-junction-forming connexin proteins. We show that the level of synchronization strongly depends on the period of ATP stimuli, as well as the connectivity between cells. Surprisingly, we find gap junctional communication between cells may destroy synchronization, resulting in a dynamically frustrated neuronal network. Our theoretical model suggests that nonlinear dynamics and a bifurcation between an excitable and periodic regime may provide key insights into understanding the experimental observations. Together, we reveal that biological neural networks display a wide array of complex collective behaviors. These behaviors are primarily influenced by external temporal signals and are further modulated by internal communications through gap junctions.

## II. RESULTS

**FIG. 1.**
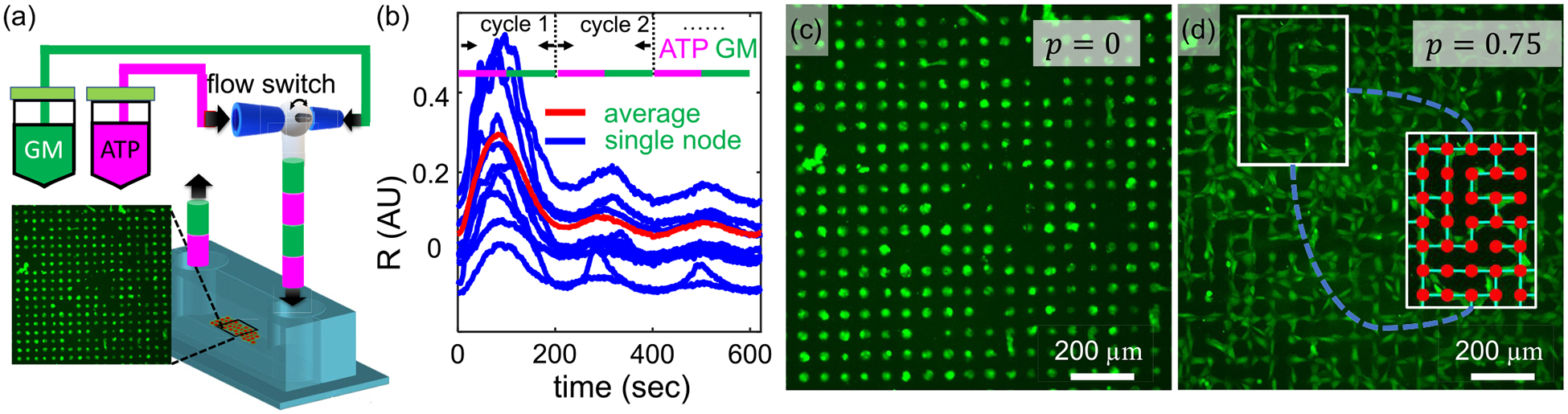
Micropatterned biological neuron networks (MBNNs) respond to temporal stimuli that trigger intracellular calcium dynamics. (a) Schematic of the experimental setup. The fluorescent image is recorded at a frame rate of 1 s per frame. (b) Fluorescent intensity of calcium imaging for an individual node (blue) and population average (red). In this example external stimuli vary between the growth medium (GM) and adenosine triphosphate (ATP) at a period of 200 s. (c) A sample fluorescent calcium image of a MBNN showing the micropatterned KTaR cell monolayer. In this example the network is disconnected with connectivity p=0. Each node has a radius of 20 μm and the edge-to-edge separation between nodes is 30 μm. (d) A sample fluorescent calcium image of a MBNN with connectivity p=75%. The nodes are bridged by micropatterned KTaR cells with a bridge width equal to 10 μm. Inset: We use automated image registration to identify effective nodes and bridges so that regions without cells attached are excluded.

In order to study the collective response to external stimuli by neuronal networks with precisely controlled spatial connectivity, we incorporate micropatterned biological neuron networks (MBNNs) to a microfluidics device (Fig. 1). In particular, the microfluidics consists of a computer-interfaced flow switch to deliver alternating growth media and ATP solution to the cell monolayer with periods ranging from 40 to 200 s [Fig. 1(a)], and with a duty cycle of 50% as characterized previously [18].

A substrate is patterned by coating a glass slide of fibrinogen surrounded by PEG polyethylene glycol (PEG) using photolithography [see Supplemental Material (SM) Secs. S1 and S2 [23]]. Fibrinogen promotes and PEG prohibits cell adhesion. We preload KTaR cells with a fluorescent calcium indicator, then pattern the cells into circular nodes of radius 20 μm. Each node typically consists of two to three cells. When exposed to periodic ATP stimuli, we measure the node response $R_{i}(t)$ by computing the average fluorescent intensity of a node i compared to its basal value [Fig. 1(b)]. To modify the spatial connectivity of the MBNNs, we arrange the nodes into a square lattice and set the edge-to-edge distance between nearest nodes to be 30 μm. Between nearby nodes, a line of single cell width is patterned to connect the node pair at probability p. When p=0, all nodes are isolated [Fig. 1(c)]. When p=0.75, each node is on average connected to three of its four neighbors [Fig. 1(d)]. We notice that at larger connectivity values cells may overgrow beyond the designed fibrinogen regions, possibly due to the resolution of our photolithography process. Therefore, to ensure accurate spatial patterning in the study, we keep the maximum connectivity to be p=0.75.

As shown previously [18], the calcium response of KTaR cells to ATP is mediated by the purigenic receptor P2X and store-operated calcium release. In the absence of chemical stimuli KTaR cells show no activities (see SM Sec. S3 for additional characterization of the cells [23]). Therefore the calcium dynamics of MBNNs are results from external ATP stimulation, rather than spontaneous fluctuations.

**FIG. 2.**
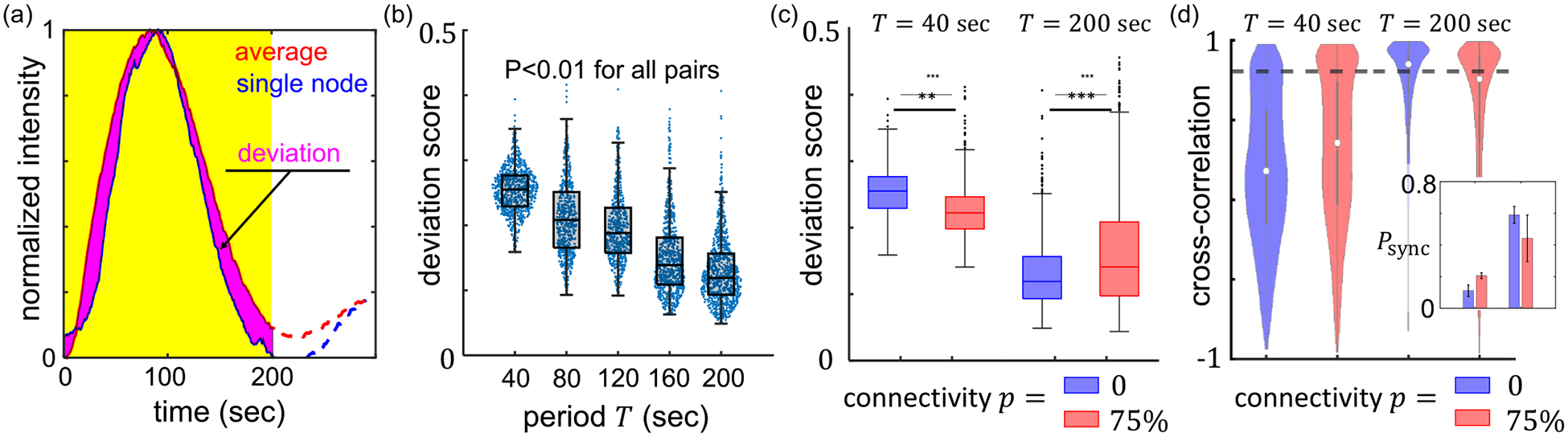
Micropatterned biological neuron networks (MBNNs) demonstrate synchronized responses to temporal stimuli with the degree of synchronization modulated by the network connectivity and period of driving signal. (a) We define the deviation score to quantify the phase difference between the calcium dynamics of the individual node and the population average. Due to the uncertainty of stimuli arrival time, we compute the deviation score in a moving window of size equal to the period and average results from the moving windows. Within the moving window, all responses are first linearly scaled to the range [0,1]. Then the deviation score is calculated as the ratio between the area of the magenta portion [labeled as deviation in (a)] to the area of the yellow rectangle. (b) At connectivity p=0, the deviation score decreases monotonically at a larger period T, indicating better synchronization when the driving signal varies slowly. For each period, 600–1000 data points are included from at least three replicating experiments. (c) The deviation scores at two driving periods (T=40s and T=200s) with network connectivity equal to 0 or 75%. The results show node-to-node communication facilitates synchronization at a short period but destroys synchronization at a long period. (d) Cross-correlation analysis between neighboring nodes shows consistent results as in (c). Here, a pair of nodes are considered as neighbors if they are bridged by cells. For disconnected networks neighboring nodes are randomly sampled nearest nodes in space. At T=40s, neighboring nodes of highly connected networks have greater cross-correlation values than those of a disconnected network. At T=200s, neighboring nodes of highly connected networks are less correlated than those of a disconnected network. We empirically consider nodes with cross-correlation greater than 0.8 as well synchronized. The threshold is indicated as the dashed line in (d). In (c) and (d) each group includes 600–1000 data points from at least three replicating experiments. Inset: Fraction of well-synchronized node pairs among all neighboring pairs ($P_{\text{sync}}$). Error bars in the inset are standard deviations from three replicating experiments. In (b) and (c), the lines and boxes show the median, upper, and bottom quartiles. In (d) the circles and lines show the median, upper, and bottom quartiles. ANOVA test is used for comparison. **: *p* < 0.01; ***: *p* < 0.001.

To characterize the synchronicity of the responses of individual nodes, we compute two quantities: the deviation score between the responses of individual nodes and the population average, and the cross-correlation coefficient between neighboring nodes. As shown in Fig. 2(a), the deviation score of a node is defined by first obtaining the normalized response $R_{i,\text{norm}}(t)$ in a moving window of width equal to the period T of the stimuli, then calculating the area (absolute value) between the curves $R_{i,\text{norm}}$ and ${\left\langle R_{i,\text{norm}}\right\rangle }_{\text{population}}$. Here, i is the index of a node and ${\left\langle R_{i,\text{norm}}\right\rangle }_{\text{population}}$ represents the average over all nodes within the field of view. To ensure fair treatment for different periods, we divide the area between the curves by the period T, thereby measuring the deviation per unit time. The deviation score is a dimensionless quantity that approaches zero when all nodes perfectly synchronize. See SM Sec. S2b for more details in the calculation [23].

We find that the deviation score continuously decreases at a longer period T even when the nodes are isolated (p=0), suggesting that cells respond more uniformly to slowly varying external stimuli [Fig. 2(b)]. The trend also holds when the nodes are connected, as shown in Fig. 2(c). Because the observation is independent of node-node communication, we attribute the synchronized response at a longer period to a decreasing impact of intercellular heterogeneity.

When external signal varies rapidly, such as the case of T=40s, intercellular communication moderately improves synchronization [Fig. 2(c)]. This is consistent with the increased cross-correlation coefficients between neighboring nodes [Fig. 2(d)]. However, when external signal varies slowly, such as the case of T=200s, MBNNs with higher connectivity exhibit asynchronous responses. Indeed, at T=200s, the average deviation score increases by 20% when connectivity increases from p=0 to p=0.75. Concurrently, fractions of well-synchronized nearest pairs—neighboring nodes with a cross-correlation coefficient higher than 0.8—decreases from 70% to 55% [Fig. 2(d) inset]. These results confirm an unexpected observation, that when driven by a slowly varying external signal the communication within a neuronal network curtails, rather than enhances, synchronization (see also SM Sec. S4 for additional results [23]). Therefore we find MBNNs to be dynamically frustrated due to the conflicting influences between external stimulation and neighboring interactions. This is distinct yet bearing similarities to other frustrated systems [24–28].

**FIG. 3.**
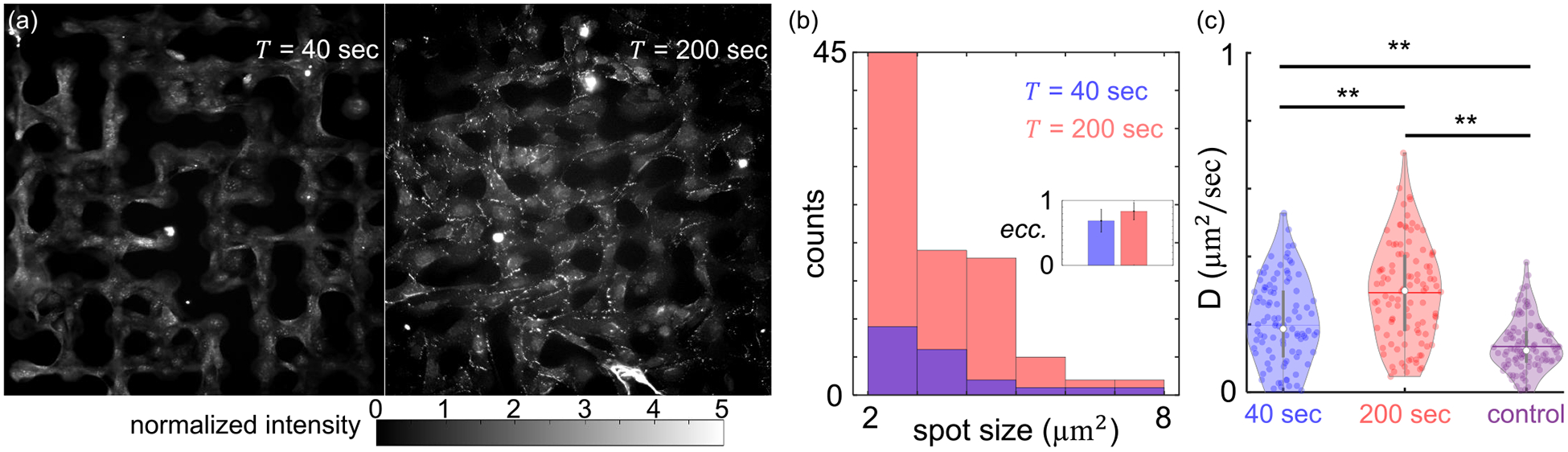
Temporal signal modulates the properties of gap junctions. (a) Immunofluorescent images of connexin 43 proteins of KTaR cells forming micropatterned biological neuron networks (MBNNs). The MBNNs are exposed to periodic ATP stimuli of period T=40s (left) and T=200s (right) before fixation and immunostaining. The image intensity has been normalized so that the background has a value of zero, and the cell cytoplasm has an mean value of 1 (see SM Sec. 2c for normalization procedures [23]). (b) Spot analysis of immunofluorescent images after binarizing the images in (a) with a threshold of 1 (the average intensity of cytoplasm). The histogram shows the distribution of spot sizes. Inset: Eccentricity of the spots. For circular spots, eccentricity is 0. For line spots, eccentricity is 1. (c) Effective intercellular diffusion coefficient D measured via FRAP (fluorescent recovery after photobleaching; see SM Sec. 2d for more details [23]). The KTaR cell monolayers are exposed to various conditions before FRAP measurements: periodic ATP stimuli of period T=40s (blue), of period T=200s (red), or zero concentration of ATP with a continuous flow of growth media (control, purple). Five repeated experiments for each condition are conducted and the diffusion coefficients from 20 consecutive frames are computed. Statistical test: N=100 for each group, and ANOVA test is used for comparison. **: *p* < 0.01.

To gain insights into the dynamics of MBNNs under temporal signals, we quantify the spatial profile of gap junctions by immunofluorescence of connexin 43. In particular, MBNNs are subject to periodic ATP stimuli for short (40 s) or long (200 s) periods for a total of 15 min. Immediately after the stimulation, MBNNs are fixed and stained using fluorescent antibodies (see SM Sec. S1 for additional details [23]). To quantify the fluorescent signals, we normalize the images by first subtracting the background, then scale the intensity such that the basal level intensity of cytoplasm is one (see SM Sec. S2 for an image analysis [23]). The normalized immunofluorescent images shown in Fig. 3(a) clearly demonstrate the different gap junction expressions under rapid and slow temporal signals. When the external signal varies rapidly (T=40s), connexin 43 appears to be mostly diffusive throughout the cells. However, when the external signal varies slowly (T=200s), connexin 43 forms punctate plaques at the cellular junctions. Quantitative analysis of the bright spots further confirms the visual observations. At T=200s, connexin 43 assembles into more densely populated, more elongated structures compared with the case of T=40s [Fig. 3(b)]. Abolishing calcium dynamics by treating the cells with thapsigargin while maintaining ATP stimuli removes junction plaques, confirming that the slow oscillation (T=200s) of intracellular calcium enhances gap junction formation (see SM Sec. S3 for additional details [23]).

In addition to morphological characterizations, we have also tested if the conductivity of gap junctions can be modulated by external temporal signals. To this end, we subject confluent monolayers of KTaR cells to periodic ATP stimuli and then measure the effective intercellular diffusion coefficients using fluorescent recovery after photobleaching (FRAP) approach we developed previously [16] (see also SM Sec. S2 for details [23]). As shown in Fig. 3(c), the diffusion coefficient D of a cell monolayer depends strongly on the period of ATP stimuli. At T=40s, the average diffusion coefficient is approximately 0.2 μm^2^/s. When the temporal signal slows down to T=200s, the average diffusion coefficient increases by 50% to 0.32 μm^2^/s. As a control, we have also conducted FRAP experiments without ATP stimuli, such that cell monolayers are subject to constant flow of growth media. The resulted diffusion coefficient is moderately smaller compared with the case of rapid stimuli (T=40s), and is less than half of the value for slow stimuli (T=200s). Furthermore, both immunofluorescent imaging and electrophysiology measurement suggest that no synapses are formed within the MBNNs (SM Sec. S3 [23]). Taken together, morphological [Figs. 3(a) and 3(b)] and functional [Fig. 3(c)] studies confirm that intercellular communication is enhanced when the external temporal signal slows down.

Our experimental results suggest that increased coupling between cells may contribute to the destruction of synchronization in MBNNs. To investigate the putative underlying mechanism, we develop a computational model of coupled excitable neurons. Previous theoretical [29–32] and experimental [33,34] work has shown that coupling can lead to desynchronization among oscillatory responses. However, in our system, cells do not oscillate intrinsically. Rather, they are periodically excited by an external signal. To our knowledge, desynchronization of periodically driven, coupled, excitable responses remains unexplored. In fact, the observation of desynchronization in this case would be more notable, as it would suggest that the desynchronizing effect of coupling can overpower the synchronizing effect of periodic driving.

Previous work identifies proximity to a particular type of bifurcation, called the saddle-node homoclinic orbit (SNHO) bifurcation, as responsible for desynchonization between oscillators [32,34]. Because this bifurcation separates an oscillatory regime from an excitable regime, here we ask whether a system in the excitable regime can still exhibit desynchronization when driven periodically.

A well-established neuron model that exhibits the SNHO bifurcation is the Morris-Lecar model [35–37], which reads

(1) $$\frac{dV}{dt}=\frac{1}{C_{\text{M}}}\left[I_{\text{ext}}-\left(V-E_{\text{Ca}}\right)g_{\text{Ca}}M_{\infty }(V)\right.\left.-\left(V-E_{\text{K}}\right)g_{\text{K}}N-\left(V-E_{\text{L}}\right)g_{\text{L}}\right]\equiv F\left(V,N\right),$$

(2) $$\frac{dN}{dt}=\frac{N_{\infty }(V)-N}{\tau_{N}(V)}\equiv G\left(V,N\right),$$

with

(3) $$M_{\infty }(V)=\frac{1}{2}\left[1+\text{tanh}\left(\frac{V-U_{\text{Ca}}}{W_{\text{Ca}}}\right)\right],\;N_{\infty }(V)=\frac{1}{2}\left[1+\text{tanh}\left(\frac{V-U_{\text{K}}}{W_{\text{K}}}\right)\right],\;\tau_{N}(V)=\frac{1}{\phi }\text{sech}\left(\frac{V-U_{\text{K}}}{2W_{\text{K}}}\right).$$

Here, V is the voltage across the membrane, which changes due to three channels: a calcium channel, a potassium channel, and a leak channel [Eq. (1)]. N is the activation variable corresponding to the potassium channel, which relaxes to the voltage-dependent value $N_{\infty }$ on a timescale $\tau_{N}$ [Eq. (2)]. In contrast, calcium depends on voltage instantaneously via $M_{\infty }$. The physiological interpretation of the parameters and their standard values used across previous studies [29,36,37] are given in Table I. In particular, with these values, the SNHO bifurcation occurs at an external current value of approximately $I_{\text{ext}}=40\;\mu \text{A}/{\text{cm}}^{2}$, with excitable dynamics occurring below this value and oscillatory dynamics occurring above this value. Therefore, to investigate the possibility of desynchronization, we use the value $I_{\text{ext}}=39.5\;\mu A/{\text{cm}}^{2}$, corresponding to excitable dynamics below, but close to, the bifurcation.

Gap junctions introduce a linear voltage coupling between cell i and each of its neighbors j,

(4) $$\frac{dV_{i}}{dt}=F\left(V_{i},N_{i}\right)+\sum_{j}\left(V_{j}-V_{i}\right)\gamma_{ij},\;\frac{dN_{i}}{dt}=G\left(V_{i},N_{i}\right),$$

where F and G are the dynamics defined in Eqs. (1) and (2), and $\gamma_{ij}$ is the coupling strength. As in the experiments, for a given cell configuration, we allow each pair of cells to be connected with probability p. Thus, for each pair, we set $\gamma_{ij}=\gamma$ with probability p, and $\gamma_{ij}=0$ otherwise, where γ is a constant. We model the periodic stimulation of ATP with driving period T by taking

(5) $$V_{i}\to V_{i}+\Delta V_{i}\;\text{when}\;t=nT,$$

for all cells, where n is an integer. We set $\Delta V_{i}$ on average to the minimum voltage required to induce an excitation in an isolated cell, which we find is 5 mV.

**FIG. 4.**
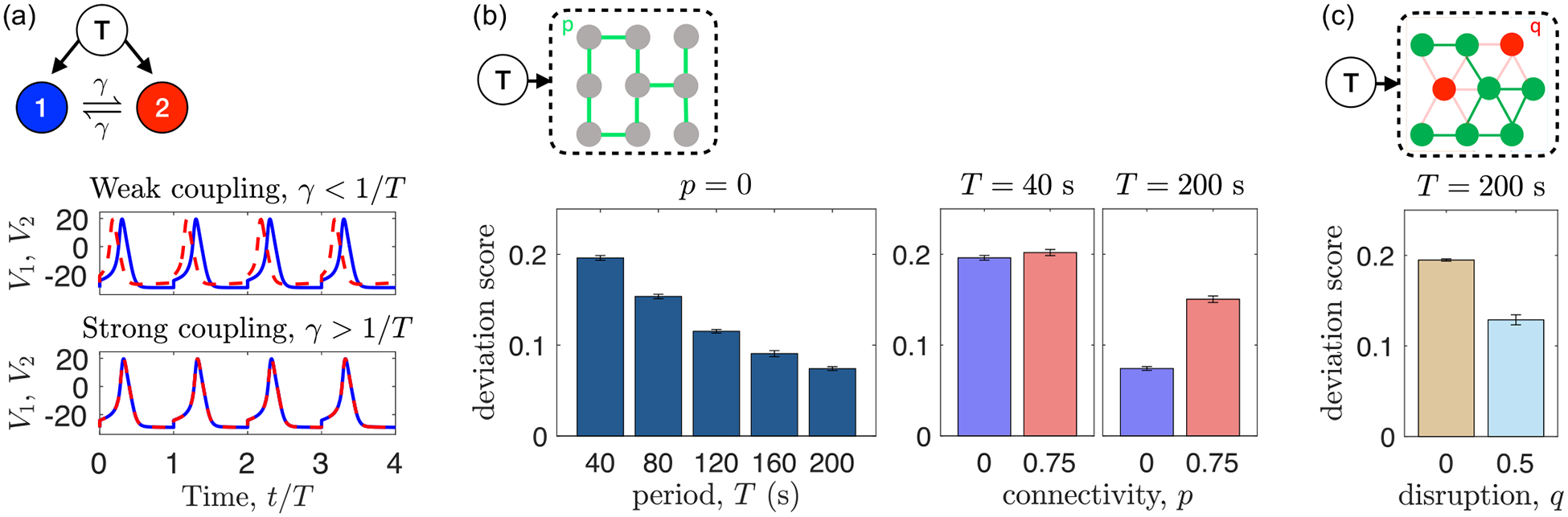
A computational model demonstrates the underlying mechanisms that lead a communicating neural network to be dynamically frustrated. (a) Two excitable cells with coupling strength γ driven externally with period T=80s. Top: Weak coupling (γ=0.25/T) desynchronizes the dynamics. Bottom: Strong coupling (γ=4/T) synchronizes the dynamics. Here, ΔV=5mV, and dynamics are shown after a relaxation time of 16T. (b) 18 × 18 square lattice of cells connected with probability p and driven with period T. Left: When p=0, the deviation score decreases with T. Right: At T=40s, the deviation score remains unchanged with p, whereas at T=200s, the deviation score increases with p. (c) 18 × 18 triangular lattice of cells disrupted (no connections) with probability q and driven with period T=200s. The deviation score decreases with q. In (b) and (c), γ=aT with $a=10^{-5}{\;\text{s}}^{-2}$, $\Delta V_{i}$ are drawn uniform-randomly from 0 to 10 mV, and the deviation score is averaged over all cells, over 10 periods (after relaxation time 10T), and over 10 random samplings, with error bars given by the standard deviation. Throughout, all parameters are as in Table I.

Figure 4(a) shows the dynamics of our model with two excitable cells, coupled with certainty p=1 and strength γ, and driven with period T. The cells have identical parameters, meaning that without coupling (γ=0), they respond in phase with the driving and thus have identical dynamics. However, we see in the top panel of Fig. 4(a) that with weak coupling (0<γ<1/T), the dynamics desynchronize. In contrast, we see in the bottom panel of Fig. 4(a) that with strong coupling (γ>1/T), the dynamics synchronize. This effect is similar to the previously observed antiphase desynchronization induced by the weak coupling of intrinsic oscillators [29–32], but here observed with periodically driven excitable responders. Here, the desynchronization is weaker than antiphase because it competes with the tendency to be in phase with the external driver. We conclude that the weak coupling of driven excitable systems can induce desynchronization.

Next, we ask whether this model can explain the experimental observations in Fig. 2. To do so, we consider an 18 × 18 square lattice of cells, which is comparable in size to the micropatterned grid. We introduce heterogeneity in the cells’ ATP responses by drawing their $\Delta V_{i}$ values uniform-randomly from 0 to 10 mV (the results in Fig. 4 do not depend qualitatively on this range). We account for the observation that coupling increases with period T (Fig. 3) by setting γ=aT (the results in Fig. 4 do not depend qualitatively on the value of a so long as weak coupling holds, γ<1/T; for the largest period T=200s, this implies $a<1/T^{2}=2.5\times 10^{-5}{\;\text{s}}^{-2}$). First, we consider no connectivity (p=0), and we see in the left panel of Fig. 4(b) that the deviation score decreases with the period T, consistent with the experiments [Fig. 2(b)]. Evidently, slower driving reduces heterogeneity among cell responses, as hypothesized experimentally. Then, we compare unconnected (p=0) and connected (p=0.75) responses to fast (T=40s) and slow (T=200s) driving, as shown in the right panel of Fig. 4(b). Consistent with the experiments [Fig. 2(c)], we see that the deviation score decreases with period at either connectivity, and increases with connectivity at T=200s. In the model the deviation score remains unchanged with connectivity at T=200s, whereas in the experiments it decreases, but this decrease may not be definitive, as we discuss later. Taken together, we conclude that the model, accounting for the experimental observation in Fig. 3, can explain the experimental data in Fig. 2.

Finally, we use the model to make a new prediction. Specifically, we ask whether desynchronization would still occur in a confluent monolayer, without the controlled structure of the micropatterned grid. To address this question, we consider a co-culture of communicating and noncommunicating cells, which we have the ability to produce experimentally, described shortly. As we have shown previously [18], in a confluent monolayer most cells have six nearest neighbors. Therefore, we replace the square lattice in our simulations with the six-neighbor triangular lattice. Simulations are run similarly to the square lattice but instead of an edge probability p, each cell has a probability q of being noncommunicating. If a cell is noncommunicating, it is not coupled to any of its six neighbors (i.e., $\gamma_{ij}=0$ for all j neighbors of noncommunicating cell i). We see in Fig. 4(c) that, due to the desynchronizing effects of the coupling, the deviation score is predicted to be larger in the regular network (q=0, entirely communicating cells) than in the disrupted network (q=0.5, a co-culture of half communicating, half noncommunicating cells).

**FIG. 5.**
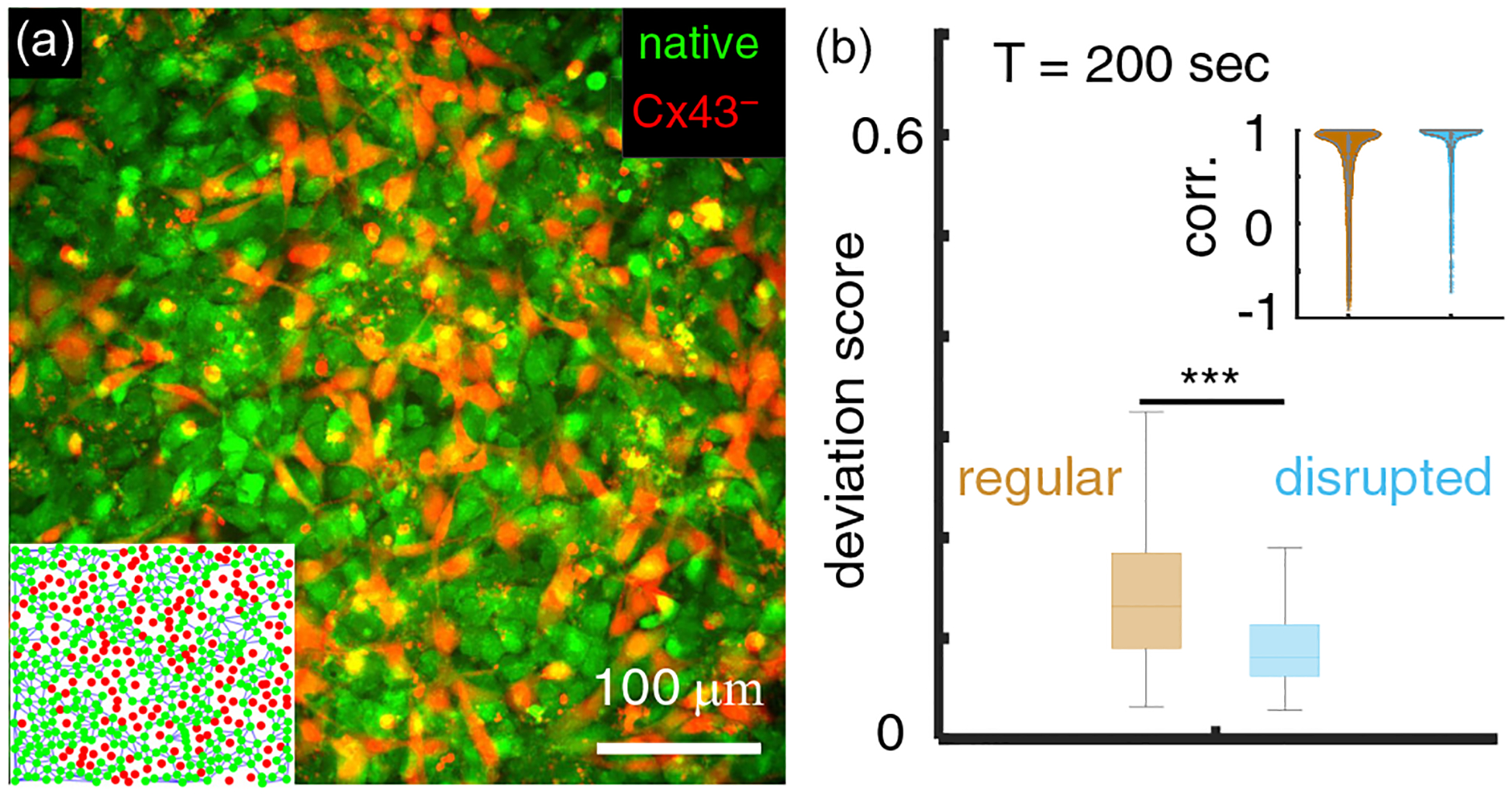
Experimental verification of model predictions demonstrate the nonlinear dynamics of cells may contribute to the dynamic frustration of biological neural networks. (a) Fluorescent image of a KTaR cell monolayer with disrupted intercellular communication. The disrupted multicellular networks consist of 50% native KTaR cells [green, labeled as native in (a)] and 50% KTaR cells with a knocked down expression of connexin 43 [red, labeled as $\text{Cx}43^{-}$ in (a)]. Inset: On average each cell has six nearest neighbors where three neighbors are communication-deficient $\text{Cx}43^{-}$ cells. Neighboring native cells (green dots) are linked by lines. (b) When exposed to slowly varying ATP stimuli (T=200s) the cell monolayers with disrupted communication show a smaller deviation score compared with regular monolayers formed by native KTaR cells. Inset: The cross-correlation coefficient of neighboring native cells in regular and disrupted KTaR monolayers. In (b) each group includes ~700 data points from three replicating experiments and the ANOVA test is used to compare the regular and disrupted monolayers. Abbreviations: corr.: cross-correlation coefficient.

To test the model prediction in Fig. 4(c), we constructed a subcloned KTaR cell line possessing disrupted intercellular communication. In particular, we employed a CRISPR-CAS9 strategy (Santa Cruz Biotechnology) to generate a KTaR cell line ${\text{CX}43}^{-}$ with the connexin 43 gene (*gja1*) being knocked out (see SM Fig. 5 for a characterization of ${\text{CX}43}^{-}$ [23]). When mixing native and ${\text{CX}43}^{-}$ cells to form a confluent monolayer, the effective connectivity of the multicellular network is reduced. As an example, in this study we focus on communication-disrupted monolayers consisting of equal amounts of native KTaR cells [green in Fig. 5(a)] and ${\text{CX}43}^{-}$ cells [red in Fig. 5(a)]. Since most cells have six neighbors, the mixture can be considered a network on a triangular lattice.

We compare the dynamics of regular cell monolayers, which consist of native KTaR cells only, and the dynamics of communication-disrupted monolayers, which consist of a 1:1 ratio of native KTaR cells and $\text{CX}43^{-}$ cells. When the external temporal signal varies slowly, the gap junctional coupling between cells is strong and our model predicts that disrupting communication between cells improves the synchronization [Fig. 4(c)]. This is indeed the case in our experimental observation. As shown in Fig. 5(b), when exposed to ATP stimuli of a period T=200s, communication-disrupted monolayers have an average deviation score of 0.08, which is nearly half of the deviation score of regular monolayers. Consistently, the nearest-neighbor cells in a communication-disrupted monolayer demonstrate higher cross-correlation coefficients than neighboring cells in a regular monolayer. Together, these experimental results show that the nonlinear excitable dynamics exhibited by neuronal cells plays an important role in contributing to the dynamic frustration of biological neural networks.

## III. CONCLUSION AND DISCUSSION

While a single cell’s response to temporal signals is regulated by the kinetics of intracellular pathways [38–40], a multicellular system relies on intercellular communications to generate collective sensory responses [41]. In this article, we examine the calcium dynamics of KTaR cells, a neuronal cell line we derived from KNDy neurons within the arcuate nucleus of an adult female mouse. KTaR cells express connexin-43 proteins *in vitro*, which form gap junctions to mediate communications between adjacent cells. We show that the nonlinear, excitable dynamics of KTaR cells are coupled via gap junctions such that the neural network exhibits rich collective dynamics when exposed to rhythmic ATP stimuli. In particular, we combine microfluidics and substrate engineering in order to create micropatterned biological neural networks (MBNNs) whose connectivity can be precisely controlled (Fig. 1). To quantify the synchronicity of calcium dynamics in a MBNN excited by temporal signals, we compute the (individual-to-population) deviation score and (individual-to-individual) cross-correlation coefficient, which yield consistent results in all our tests. The temporal signals consist of an alternate ATP solution, a common neurotransmitter, and pure growth medium, at varying periods.

When exposed to slower temporal signals, as in the case of increasing the period of ATP stimuli, we find that individual nodes in a MBNN become increasingly synchronized (Fig. 2). Because the phenomenon is also present in isolated nodes from MBNNs of zero connectivity (p=0), a putative explanation is that a longer period suppresses the effects of intercellular heterogeneity [42]. Interestingly, at a large period (T=200s), poorly connected neural networks (p=0) outperform highly connected ones (p=0.75) in synchronicity metrics (Fig. 2), suggesting that gap junctions potentially impede communicating cells from synchronization. Indeed, as the period of ATP stimuli increases, we observe an elevation in the expression level of the gap junctions, which is accompanied by a concurrent rise in the speed of molecule exchange between cells (Fig. 3). This result is also consistent with previous reports that the information exchange between neuronal cells increases when external stimuli vary at slower rates [18]. Together, our results show that slow temporal signals enhance the gap junctional communication between KTaR cells. However the enhanced communication restrains, rather than facilitates, the synchronized response of a MBNN to external stimuli. Interestingly, previous reports also show that the conduction velocity in cardiac tissues does not monotonically grow with gap junction coupling. Instead, strong coupling amplifies the phase delay between neighboring cells [43,44], similar to the results we report here.

In order to understand the role of communication in synchronizing sensory responses of a neural network, we devise a computational model and show that the nonlinear dynamics of individual units depends nontrivially on the interplay between external driving and linear coupling in collective dynamics. The model reproduces the experimental observations that increasing the driving period increases synchronization, and that increasing the coupling between cells decreases synchronization (Fig. 4). The model offers an explanation: Coupling desynchronizes cells because the dynamics are near a saddle-node homoclinic orbit (SNHO) bifurcation, which is known to promote desynchronization among intrinsic oscillators [29–34], and here we show that it promotes desynchronization among periodically driven excitable responders. The model also makes a prediction—that co-cultures of communicating and noncommunicating cells will be more synchronized than a culture of communicating cells—which is confirmed by our experiments (Fig. 5).

**FIG. 6.**
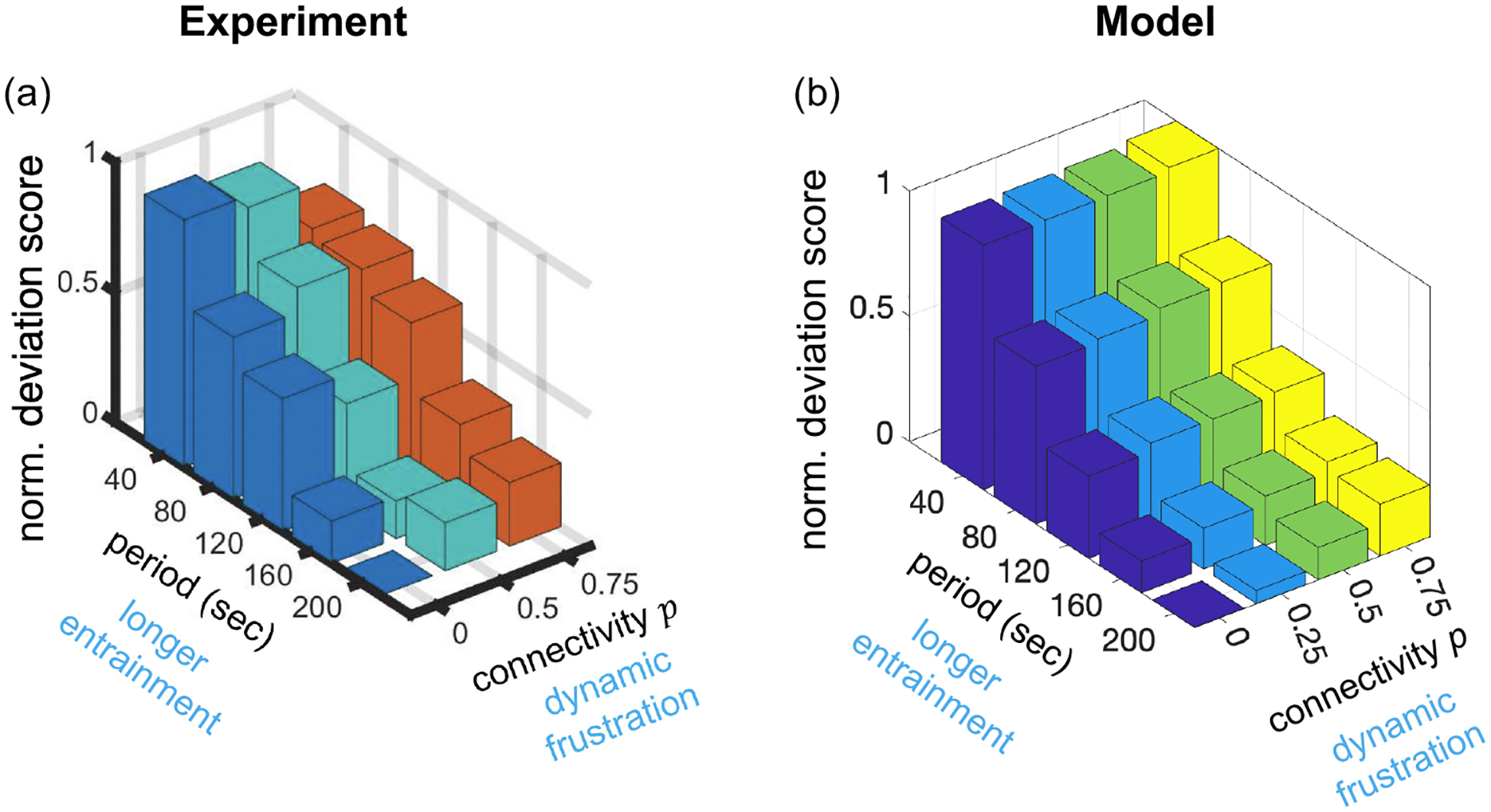
Dynamics of communicating biological neural networks are controlled by external temporal signals (period) and internal connectivity. Our experiments (a) show, and our computational model (b) confirms, that cell-to-cell deviations decrease with period and increase with connectivity. The model suggests that the former result is because longer driving periods allow a more synchronous response despite cell-to-cell heterogeneity, an effect we term longer entrainment; and that the latter result is because coupling promotes desynchronization between neighbors that must be resolved among all connections, an effect we term dynamic frustration. Model parameters are as in Fig. 4(b) with $a=0.5\times 10^{-5}{\;\text{s}}^{-2}$.

Taken together, the model suggests that the experimental data can be summarized along two key axes, as shown in Fig. 6: (1) Cells deviate less with increasing driving period because longer periods allow more time to entrain to the stimulus. (2) Cells deviate more with increasing connectivity because coupling promotes desynchronization between neighbors. The network must resolve this conflict across all connections, which leads to frustration in single cell dynamics. We remark that in the experiments the deviation decreases with connectivity at very fast periods [T=40s, Fig. 6(a)], whereas in the model the deviation remains constant [Fig. 6(b)]. However, by T=80s in the experiments, this dependence is already reversed. Therefore, we conclude that longer entrainment and dynamic frustration are the dominant effects that govern the networked response to periodic stimuli in this system.

In mice and other mammals, KNDy neurons are critical for pubertal progression and fertility, and represent hypothalamic neurons capable of pulsatile kisspeptin release. Mechanisms underlying pulse generation are incompletely characterized, and may involve both neuropeptide release and gap junctional communication. Results elucidating cellular synchronization and communication strategies among these neurons will provide insight into these mechanisms. For instance, in the brain, gap junctional coupling between KNDy neurons is often physically disrupted by axonal length, interneurons, and glia. Our results suggest that the synchronization between KNDy neurons may benefit from such a spatial arrangement.

Biological neural networks are the building block of cognition, learning, and intelligence [45,46]. Just as neural networks in computer science, the functions of biological neural networks rely on their ability to rewire the interneuron interactions [47,48]. Our results show that the property of gap-junction-mediated neuronal cell interactions can be significantly tuned by an external temporal signal, underscoring the importance of nonlinear neuronal dynamics in controlling collective behaviors. We envision future research to extend the collective sensory responses of micropatterned neural networks to build functional and adaptive neural circuits.

## IV. METHODS

See the SM Appendix for details of cell culture, microscopy, and image analysis [23]. The statistical analysis and computer simulations are performed with matlab (Math-Works^®^).

## Supplementary Material

SI

## Figures and Tables

**TABLE I.** Parameters used in Eqs. (1)–(3). All but the last two are standard values used for the Morris-Lecar model in previous studies [29,36,37]. The values of ϕ and $I_{\text{ext}}$ are chosen to place the system in the excitable regime close to the SNHO bifurcation [36].

$C_{\text{M}}$	Membrane capacitance	$20\;\mu \text{F}/{\text{cm}}^{2}$
$g_{\text{Ca}}$	Calcium channel conductance	$4\;\text{mS}/{\text{cm}}^{2}$
$g_{\text{K}}$	Potassium channel conductance	$8\;\text{mS}/{\text{cm}}^{2}$
$g_{\text{L}}$	Leak channel conductance	$2\;\text{mS}/{\text{cm}}^{2}$
$E_{\text{Ca}}$	Calcium equilibrium potential	120mV
$E_{\text{K}}$	Potassium equilibrium potential	-80mV
$E_{\text{L}}$	Leak equilibrium potential	-60mV
$U_{\text{Ca}}$	Calcium midpoint potential	-1.2mV
$U_{\text{K}}$	Potassium midpoint potential	12mV
$W_{\text{Ca}}$	Calcium steepness potential	18mV
$W_{\text{K}}$	Potassium steepness potential	17.4mV
ϕ	Potassium response rate	$0.184{\;\text{s}}^{-1}$
$I_{\text{ext}}$	External current	$39.5\;\mu \text{A}/{\text{cm}}^{2}$

## Data Availability

The data that support the findings of this article are openly available [49].

## References

[1] Mitchell A, Wei P, and Lim WA, Oscillatory stress stimulation uncovers an Achilles heel of the yeast MAPK signaling network, Science 350, 1379 (2015). 26586187 10.1126/science.aab0892 PMC4721531

[2] Rein P, Becker NB, Ouldridge TE, and Mugler A, Fundamental limits to cellular sensing, J. Stat. Phys 162, 1395 (2016).

[3] Wascher CAF, Heart rate as a measure of emotional arousal in evolutionary biology, Philos. Trans. R. Soc. B 376, 20200479 (2021). 10.1098/rstb.2020.0479 PMC8237168 34176323

[4] Sanchez RE, Kalume F, and de la Iglesia HO, Sleep timing and the circadian clock in mammals: Past, present and the road ahead, Seminars Cell & Develop. Biol 126, 3 (2022). 10.1016/j.semcdb.2021.05.034 PMC8800593 34092510

[5] Tang W, Das A, Pegoraro AF, Han YL, Huang J, Roberts DA, Yang H, Fredberg JJ, Kotton DN, Bi D, and Guo M, Collective curvature sensing and fluidity in three-dimensional multicellular systems, Nat. Phys 18, 1371 (2022). 40458282 10.1038/s41567-022-01747-0 PMC12129127

[6] Saw TB, Doostmohammadi A, Nier V, Kocgozlu L, Thampi S, Toyama Y, Marcq P, Lim CT, Yeomans JM, and Ladoux B, Topological defects in epithelia govern cell death and extrusion, Nature (London) 544, 212 (2017). 28406198 10.1038/nature21718 PMC5439518

[7] Dhar J, Thai ALP, Ghoshal A, Giomi L, and Sengupta A, Self-regulation of phenotypic noise synchronizes emergent organization and active transport in confluent microbial environments, Nat. Phys 18, 945 (2022).

[8] Eidi Z, Khorasani N, and Sadeghi M, Reactive/less-cooperative individuals advance population’s synchronization: Modeling of dictyostelium discoideum concerted signaling during aggregation phase, PLoS One 16, e0259742 (2021). 34793512 10.1371/journal.pone.0259742 PMC8601469

[9] Agladze NN, Halaidych OV, Tsvelaya VA, Bruegmann T, Kilgus C, Sasseb P, and Agladze KI, Synchronization of excitable cardiac cultures of different origin, Biomater. Sci 5, 1777 (2017). 28643840 10.1039/c7bm00171a

[10] Glennon M, Keane MA, Elliott MA, and Sauseng P, Distributed cortical phase synchronization in the EEG reveals parallel attention and working memory processes involved in the attentional blink, Cereb. Cortex 26, 2035 (2015). 25750255 10.1093/cercor/bhv023

[11] Nitsan I, Drori S, Lewis YE, Cohen S, and Tzlil S, Mechanical communication in cardiac cell synchronized beating, Nat. Phys 12, 472 (2016).

[12] Czajkowski BM, Batista CA, and Viana RL, Synchronization of phase oscillators with chemical coupling: Removal of oscillators and external feedback control, Physica A 610, 128418 (2023).

[13] Chartrand T, Goldman MS, and Lewis TJ, Synchronization of electrically coupled resonate-and-fire neurons, SIAM J. Appl. Dyn. Syst 18, 1643 (2019). 33273894 10.1137/18m1197412 PMC7709966

[14] Bressloff PC and Lai Y, Stochastic synchronization of neuronal populations with intrinsic and extrinsic noise, J. Math. Neurosci 1, 2 (2011). 22656265 10.1186/2190-8567-1-2 PMC3280892

[15] Popovych OV and Tass PA, Control of abnormal synchronization in neurological disorders, Front. Neurol 5, 268 (2014). 25566174 10.3389/fneur.2014.00268 PMC4267271

[16] Potter GD, Byrd TA, Mugler A, and Sun B, Communication shapes sensory response in multicellular networks, Proc. Natl. Acad. Sci. USA 113, 10334 (2016). 27573834 10.1073/pnas.1605559113 PMC5027410

[17] Zamir A, Li G, Chase K, Moskovitch R, Sun B, and Zaritsky A, Emergence of synchronized multicellular mechanosensing from spatiotemporal integration of heterogeneous single-cell information transfer, Cell Syst. 13, 711 (2022). 35921844 10.1016/j.cels.2022.07.002 PMC9509451

[18] Li G, LeFebre R, Starman A, Chappell P, Mugler A, and Sun B, Temporal signals drive the emergence of multicellular information networks, Proc. Natl. Acad. Sci. USA 119, e2202204119 (2022). 36067282 10.1073/pnas.2202204119 PMC9477235

[19] Ellison D, Mugler A, Brennan MD, Lee SH, Huebner RJ, Shamir ER, Woo LA, Kim J, Amar P, Nemenman I, Ewald AJ, and Levchenko A, Cell-cell communication enhances the capacity of cell ensembles to sense shallow gradients during morphogenesis, Proc. Natl. Acad. Sci. USA 113, E679 (2016). 26792522 10.1073/pnas.1516503113 PMC4760786

[20] Glen CM, McDevitt TC, and Kemp ML, Dynamic intercellular transport modulates the spatial patterning of differentiation during early neural commitment, Nat. Commun 9, 4111 (2018). 30291250 10.1038/s41467-018-06693-1 PMC6173785

[21] Jacobs DC, Veitch RE, and Chappell PE, Evaluation of Immortalized AVPV- and arcuate-specific neuronal kisspeptin cell lines to elucidate potential mechanisms of estrogen responsiveness and temporal gene expression in females, Endocrinology 157, 3410 (2016). 27409645 10.1210/en.2016-1294

[22] Inoue N, Hazim S, Tsuchida H, Dohi Y, Ishigaki R, Takahashi A, Otsuka Y, Yamada K, Uenoyama Y, and Tsukamura H, Hindbrain adenosine 5-triphosphate (ATP)-purinergic signaling triggers LH surge and ovulation via activation of AVPV kisspeptin neurons in rats, J. Neurosci 43, 2140 (2023). 36813577 10.1523/JNEUROSCI.1496-22.2023 PMC10039743

[23] See Supplemental Material at http://link.aps.org/supplemental/10.1103/1258-cl48 for additional details of experimental procedures, data analysis, and computational models.

[24] Chalker JT, Geometrically frustrated antiferromagnets: Statistical mechanics and dynamics, in Introduction to Frustrated Magnetism (Springer, Berlin, 2011), pp. 3–22.

[25] Li Y, Du H, Wang Y, Liang J, Xiao L, Yi W, Ma J, and Jia S, Observation of frustrated chiral dynamics in an interacting triangular flux ladder, Nat. Commun 14, 7560 (2023). 37985772 10.1038/s41467-023-43204-3 PMC10662351

[26] Grason G, Perspective: Geometrically frustrated assemblies, J. Chem. Phys 145, 110901 (2016).

[27] Jia D and Muthukumar M, Topologically frustrated dynamics of crowded charged macromolecules in charged hydrogels, Nat. Commun 9, 2248 (2018). 29884894 10.1038/s41467-018-04661-3 PMC5993817

[28] Hanai R, Nonreciprocal frustration: Time crystalline order-by-disorder phenomenon and a spin-glass-like state, Phys. Rev. X 14, 011029 (2024).

[29] Han SK, Kurrer C, and Kuramoto Y, Dephasing and bursting in coupled neural oscillators, Phys. Rev. Lett 75, 3190 (1995). 10059517 10.1103/PhysRevLett.75.3190

[30] Chow CC and Kopell N, Dynamics of spiking neurons with electrical coupling, Neural Comput. 12, 1643 (2000). 10935921 10.1162/089976600300015295

[31] Nomura M, Fukai T, and Aoyagi T, Synchrony of fast-spiking interneurons interconnected by gabaergic and electrical synapses, Neural Comput. 15, 2179 (2003). 12959671 10.1162/089976603322297340

[32] Hesse J, Schleimer J-H, and Schreiber S, Qualitative changes in phase-response curve and synchronization at the saddle-node-loop bifurcation, Phys. Rev. E 95, 052203 (2017). 28618541 10.1103/PhysRevE.95.052203

[33] Vervaeke K, Lörincz A, Gleeson P, Farinella M, Nusser Z, and Silver RA, Rapid desynchronization of an electrically coupled interneuron network with sparse excitatory synaptic input, Neuron 67, 435 (2010). 20696381 10.1016/j.neuron.2010.06.028 PMC2954316

[34] Hürkey S, Niemeyer N, Schleimer J-H, Ryglewski S, Schreiber S, and Duch C, Gap junctions desynchronize a neural circuit to stabilize insect flight, Nature (London) 618, 118 (2023). 37225999 10.1038/s41586-023-06099-0 PMC10232364

[35] Morris C and Lecar H, Voltage oscillations in the barnacle giant muscle fiber, Biophys. J 35, 193 (1981). 7260316 10.1016/S0006-3495(81)84782-0 PMC1327511

[36] Tsumoto K, Kitajima H, Yoshinaga T, Aihara K, and Kawakami H, Bifurcations in Morris–Lecar neuron model, Neurocomputing 69, 293 (2006).

[37] Ermentrout B and Terman DH, Mathematical Foundations of Neuroscience, Vol. 35 (Springer, Berlin, 2010).

[38] Thomas AP, Bird GSJ, Hajnóczky G, Robb-Gaspers LD, and Putney JW, Spatial and temporal aspects of cellular calcium signaling, FASEB J. 10, 1505 (1996). 8940296

[39] Mora T and Wingreen NS, Limits of sensing temporal concentration changes by single cells, Phys. Rev. Lett 104, 248101 (2010). 20867338 10.1103/PhysRevLett.104.248101

[40] Son M, Wang AG, Keisham B, and Tay S, Processing stimulus dynamics by the NF-kB network in single cells, Exp. Mol. Med 55, 2531 (2023). 38040923 10.1038/s12276-023-01133-7 PMC10766959

[41] Japón P, Jiménez-Morales F, and Casares F, Intercellular communication and the organization of simple multicellular animals, Cells Dev. 169, 203726 (2022). 34450344 10.1016/j.cdev.2021.203726

[42] Goetz A, Akl H, and Dixit P, The ability to sense the environment is heterogeneously distributed in cell populations, eLife 12, RP87747 (2024). 38293960 10.7554/eLife.87747 PMC10942581

[43] Rohr S, Role of gap junctions in the propagation of the cardiac action potential, Cardiovasc. Res 62, 309 (2004). 15094351 10.1016/j.cardiores.2003.11.035

[44] Nowak MB, Veeraraghavan R, Poelzing S, and Weinberg SH, Cellular size, gap junctions, and sodium channel properties govern developmental changes in cardiac conduction, Front. Physiol 12, 731025 (2021). 34759834 10.3389/fphys.2021.731025 PMC8573326

[45] Chen Z, Liang Q, Wei Z, Chen X, Shi Q, Yu Z, and Sun T, An overview of in vitro biological neural networks for robot intelligence, Cyborg Bionic Syst. 4, 0001 (2023). 37040493 10.34133/cbsystems.0001 PMC10076061

[46] Kagan BJ, Kitchen AC, Habibollahi F, Khajehnejad M, Parker BJ, Bhat A, Rollo B, Razi A, and Friston KJ, In vitro neurons learn and exhibit sentience when embodied in a simulated game-world, Neuron 110, 3952 (2022). 36228614 10.1016/j.neuron.2022.09.001 PMC9747182

[47] Bennett SH, Kirby AJ, and Finnerty GT, Rewiring the connectome: Evidence and effects, Neurosci. Biobehav. Rev 88, 51 (2018). 29540321 10.1016/j.neubiorev.2018.03.001 PMC5903872

[48] Kudithipudi D, Aguilar-Simon M, Babb J, Bazhenov M, Blackiston D, Bongard J, Brna AP, Raja SC, Cheney N, Clune J, Daram A, Fusi S, Helfer P, Kay L, Ketz N, Kira Z, Kolouri S, Krichmar JL, Kriegman S, Levin M , Biological underpinnings for lifelong learning machines, Nat. Mach. Intell 4, 196 (2022).

[49] Experimental recordings of calcium dynamics are available at https://doi.org/10.6084/m9.figshare.23298476.v1; the code used to create Figs. 4, 5(a), and 5(b) is available at https://github.com/rwl23/collective_dynamics_of_frustrated_biological_neuron_networks.

