# Supplementary material for: Collective Dynamics of Frustrated Biological Neuron Networks
Source: PRX Life. Author manuscript; Available in PMC 2025 Aug 21. (PMC12366724; doi:10.1103/1258-cl48)
Supplement: SI [file NIHMS2095918-supplement-SI.pdf]

Supplementary Information

Guanyu Li[1,+], Ryan LeFebre[2,+], Alia Starman[3], Patrick Chappell[3,*], Andrew Mugler[2,*], and Bo Sun[1,*]

[1]Oregon State University, Department of Physics, Corvallis, 97331, USA
[2]Department of Physics and Astronomy, University of Pittsburgh, Pittsburgh, PA 15260
[3]Department of Biomedical Sciences, Carlson College of Veterinary Medicine, Oregon State University, Corvallis, OR 97331
[*]To whom correspondences should send to: chappelp@oregonstate.edu, andrew.mugler@pitt.edu, sunb@oregonstate.edu,
[+]these authors contributed equally to this work

ABSTRACT

S1. Additional information of experimental system

a. Fabrication of micropatterning masks

The mask template is created in a maze-like format using Matlab and consists of nodes and edges. The nodes are represented by round circles with a radius of 20 µm, while the edges are rectangular shapes with a width of 10 µm and a length of 30 µm. Subsequently, the plotted image is converted into a ".dxf" file, which is a readable format for the photolithography machine DWL66fs (Heidelberg Instruments, Germany). To print the pattern, a 4"x4"x0.06" blank quartz mask (Nanofilm, CA) is utilized. After the photolithography process following the standard protocol of the machine, the mask undergoes development, chrome etching, and PR stripping steps using PPD-455, CEP-200, and PRS-100 respectively. Finally, the mask is ready to be used.

b. Cell Culture and Micropatterning Sample Preparation

The KTaR cell line mentioned in the main text is cultured in a standard growth medium. This medium consists of Dulbecco's modified Eagle medium (DMEM, Sigma-Aldrich, MO) supplemented with 10% fetal bovine serum and 2% 100x penicillin. The cells are cultured in T-25 flasks and will be subcultured for experiments once they reach 80% to 90% confluency.

On day 0 of each micropatterning experiment, a 25 mm x 75 mm coverslip (Ibidi, Germany) is prepared using micropatterning kits (4Dcell, France) following the prescribed protocol. The coverslip is first being coated with a solution of PLL-G-PEG at a concentration of 50 µg/mL for 30 minutes at room temperature. Once coated, the coverslip is thoroughly washed with deionized (DI) water to remove any excess solution. Subsequently, the prepared coverslip is placed beneath the aforementioned mask in S1a, a homemade clamp is used to ensure close contact between the mask and coverslip. The mask and coverslip then undergo type III UV light illumination for 10 minutes using a UV/Ozone Cleaner (Bioforce Nanosciences, IL). Following this step, the coverslip is coated with Fibrinogen From Human Plasma, Alexa Fluor™ 488 Conjugate (Thermo Fisher, MA). This fibrinogen coating serves to highlight the micropatterning pattern. Finally, the coverslip is assembled with a 0.8 mm height sticky-slide I luer (Ibidi, Germany) forming a complete sample device. To ensure a leak-free seal, additional weight is applied to the assembled device by placing a heavy metal on top of the device and the device is placed on a 37°C heat plate overnight.

On day 1 of each experiment, the cell is detached from culture flasks using TrypLE Select (Life Technologies, CA) for 10 mins at 37°C, 5% humidity incubator. Following detachment, the cells are centrifuged at 123g for 5 minutes and then resuspended in the growth medium. The cell suspension is carefully pipetted into the previously prepared device described above. Approximately 300 µL of the cell suspension solution, with a concentration of approximately 5×10^5 cells/mL, is injected into the device. Subsequently, the cells are allowed to attach to the bottom surface and establish gap junctions by incubating the device at 37°C in a 5% humidity incubator for a duration of 8 to 12 hours.

After the incubation period, the fluorescent calcium indicator is prepared by using Calbryte™ 590 AM (AAT bioquest, CA) following the instructions provided: 32 µL DMSO is mixed with 50 g of Calbryte™ 590 AM to make 1.4mM solution. The final solution is composed of 8 µL of 1.4mM Calbryte™ 590 AM solution with 64 µL of 25mM probenecid, 728 µL of HHBS

and 800 µL of complete growth medium. Then, 300 µL of the final solution is added into the device and incubated for 40 – 50 mins in the incubator. To avoid apoptosis caused by dye toxicity, when conducting multiple experiments, cells are labeled one device at a time. Once the incubation is complete, the device is removed from the incubator, and the dye solution is replaced with the fresh growth medium. At this point, the device is ready for imaging.

c. Immunostaining of connexin 43 protein and F-actin

Before the immunostaining process, we prepare a solvent solution composed of PBS with 1% BSA (Sigma-Aldrich, MO) and 0.1% Triton-X100 (Sigma-Aldrich, MO). For the immunostaining, we utilize Connexin 43 Polyclonal Antibody (Invitrogen, MA) as our primary antibody and Goat anti-Rabbit IgG (H+L) Highly Cross-Adsorbed Secondary Antibody, Alexa Fluor™ 488 (Invitrogen, MA) as our secondary antibody. Both the primary and secondary antibodies are diluted or dissolved in the aforementioned solvent solution to create a 5 µg/L and 3 µg/L solution, respectively. If staining the F-actin protein, we would use Alexa Fluor™ 488 Phalloidin (Thermo Fisher, MA) which was firstly dissolved in DMSO and then diluted to 0.33 µM in 1x PBS.

Before the immunostaining process, MBNNs are treated with different periods of ATP stimulation with or without thapsigargin depending on the experimental condition we want to investigate. When administrating thapsigargin to MBNNs, we follow our previous protocol[1] where we add 1 µM of thapsigargin into the ATP solution and growth medium that are used for alternative perfusion. The fixation, permeabilization, and blocking process is performed using the Image-iT™ Fixation/Permeabilization Kit (Thermo Fisher, MA). The entire process follows the protocol provided by Thermo Fisher, with the exception that during the washing and blocking steps, the device is placed on the SK-o180-S shakers (Onilab, CA) set to a rotation speed of 80 rotations per minute. This ensures more effective removal of excess solution and proper blocking of the sample.

When staining the F-actin, 300 µL of the Phallodin stock staining solution at the right concentration will be added to the device, and the device was incubated at room temperature for 20 minutes in the dark. When staining the connexin 43 protein, 300 µL of the primary antibody solution was added to the device and stored overnight at 4°C in the refrigerator. After primary antibody staining, the device is washed three times with PBS (5 minutes each time), with the device placed on the shaker during the entire washing process. Next, 300 µL of the secondary antibody solution was added to the device, and the device was incubated at room temperature for 95 minutes in the dark. After staining the target protein, the device was washed with PBS in the dark and ready for imaging.

d. Preparing communication-disrupted monolayer

The communication-disrupted monolayer is prepared in the PDMS device described in our previous research[1]. Prior to subculture, connexin 43 knock-out cells are labeled with CellTracker™ Green CMFDA Dye (Thermo Fisher, MA). Each vial of the dye is first dissolved in 100 µL of DMSO, and then the solution is further diluted to a concentration of 5 µg/mL with a total volume of 6 mL in growth medium. This solution is added to a T-25 flask containing connexin 43 knock-out cells at approximately 80%-90% confluency and the flask is incubated at 37°C in a 5% humidity incubator for 30 minutes. Following this, both the connexin 43 knock-out KTaR cell flask and a regular KTaR cell flask are subcultured simultaneously using the aforementioned subculture protocol in S1b. The cell suspension solutions from both flasks are then mixed together to achieve a final solution with a 1:1 ratio of connexin 43 knock-out cells to regular KTaR cells. Then the solution is diluted with the growth medium to the desired density (around 2000 cells/mm^2) and then injected into the PDMS device. The PDMS device is subsequently placed in the incubator for 8-12 hours to allow the cells to attach to the bottom and form gap junctions. After the incubation period, the communication-disrupted monolayer is labeled with a fluorescent calcium indicator, following a similar procedure as described earlier but using Calbryte™ 590 AM (AAT bioquest, CA) to avoid interference with the CellTracker™ fluorescent dye. Finally, the cells are ready for imaging.

e. Fluorescent recovery after photobleaching (FRAP) experiments

The cell suspension with density around 1.87×10^6 cells/mL is added to the ibidi device composed of an unpatterned coverslip and a 0.8 mm height sticky-slide I luer (Ibidi, German) to prepare a confluent cell monolayer. On day 1 of the experiment, cells are labeled with CellTracker™ Green CMFDA Dye (Thermo Fisher, MA) using the same recipe and procedure we described in S1d. Then cells are exposed to 15 mins of periodic ATP stimuli with short (40s) or long periods (200s). Right after the stimulation, the device is sent to a Leica DMI-8 microscope to perform FRAP experiments.

During the FRAP experiments, the fluorescent image of the monolayer is recorded at 1 Hz for 3 seconds before the photobleaching. Then a 80 µm circular area is chosen as the photobleaching area and 445 nm laser is illuminated for 30-60 seconds to photo bleach the area. Afterward, the recovery behavior from photobleaching is characterized by recording the fluorescent image of the monolayer at 1/30 Hz for at least 1500 seconds.

S2. Additional information of data analysis

a. Cross-correlations

The cross-correlation between node-to-node pairs or node-to-population is computed for each cycle, using a zero lag between the intensity data of the pair for calculation. The choice of node-to-node pairs is based on the pattern of the neuron network and the real connection for each node. Here is the formula used for calculating the cross-correlation assume we have node A with intensity I_A and node B with intensity $I_B(t)$:

$$\text{cross correlation} = \frac{< (I_A - \overline{I_A})(I_B - \overline{I_B}) >}{std(I_A - \overline{I_A}) * std(I_A - \overline{I_A})} \tag{1}$$

where $\overline{I_A}$ and $\overline{I_B}$ refer to the average of intensity for node A and node B respectively. In the case of calculating the cross-correlation between the individual node and the population, we use the same formula to calculate the cross-correlation between individual node intensity data and the population intensity data which is the average intensity across all nodes.

b. Computing deviation score

The deviation score is determined by comparing the raw intensity data of each node to the population intensity data. To calculate the deviation score, we employ a moving window approach with a window size equal to the period of ATP stimulation. The window starts at the time when ATP arrives and moves until the right end of the window reaches the end of our experimental recording. The time step between consecutive windows is 1 second. Within each moving window, both the single node and population intensity data are normalized to the range [0, 1] using the following formula:

$$I_{norm}(t) = \frac{I(t) - I_{min}}{I_{max} - I_{min}} \tag{2}$$

Where $I_{norm}(t)$ refers to the normalized intensity data, I_{min} and I_{max} refers to the minimum and maximum intensity data within the moving window.

After the normalization process, we compute the deviation score per window K_{i,t_0}, where i is the node index, and t_0 is the starting frame of the window. K_{i,t_0} is the ratio of the non-overlapping area to the total window area and is calculated using the following formula:

$$K_{i,t_0} = \sum_{t=0}^{period} \frac{|I_{\text{norm, node i}}(t + t_0) - I_{\text{norm, population}}(t + t_0)|}{period} \tag{3}$$

where the $I_{\text{norm, node i}}(t)$ and $I_{\text{norm, population}}(t)$ refer to the normalized intensity for a single node i and population respectively.

The deviation score for node i is computed by averaging K_{i,t_0} over starting frame t_0: $K_i = \langle K_{i,t_0} \rangle_{t_0}$.

Finally the deviation score K for a typical experiment is defined as the population average of K_i for all nodes in the field of view.

c. Image processing of the immunofluorescent images

First, we calculate the background brightness intensity data for background areas that are not occupied by cells and for cell baseline regions where connexin 43 is not aggregated. We calculated the cell non-occupied brightness intensity BI_{empty} or the cell baseline brightness intensity $BI_{baseline}$ by calculating the average brightness intensity for multiple empty background regions or cell baseline regions. Next, we normalize the image using these two brightness intensity values, ensuring that the raw brightness intensity BI within the range of $[BI_{empty}, BI_{baseline}]$ is mapped to the new range of [0, 1]:

$$BI_{norm} = \frac{BI - BI_{empty}}{BI_{baseline} - BI_{empty}} \tag{4}$$

For values of BI_{norm} greater than 5, we treat them as outliers and set them to a fixed value of 5. This Fig.1 shows the image after normalization.

d. Calculating effective diffusion coefficient

We quantify the monolayer's intercellular communication strength by calculating the effective diffusion coefficient from previous research[2]. We modify the formula to match our circular photobleaching area. In our calculation, the field of view is focused on a 120 µm x 120 µm square region enclosing the photobleached circular region at the center(Fig.2A). Thus, our formula used to calculate the diffusion coefficient would be:

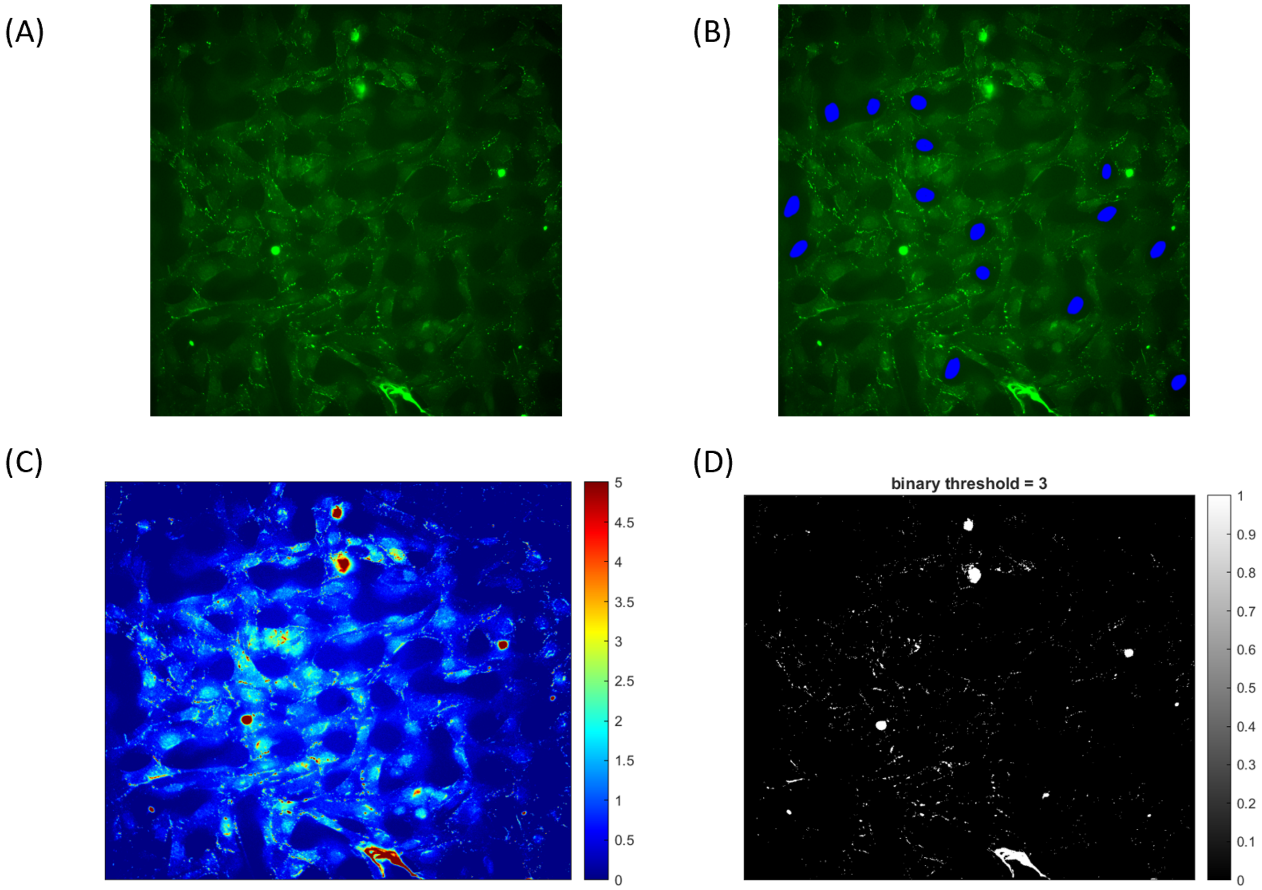

Figure S1. Illustration of the immunofluorescent imaging and image analysis of Connexin 43. (A) Raw image from the immunostaining experiment with an edge probability of 0.75 and after a 200-second period of ATP stimulation for 15 minutes. (B) Dotted regions represent the cell non-occupied area used to calculate BI_{empty}. The blue spots indicate the regions and the brightness intensity for each region is obtained by averaging the intensities of all pixels within the blue area. (C) The image was plotted using the normalized intensity data. (D) binary image after applying a binary threshold on (C)

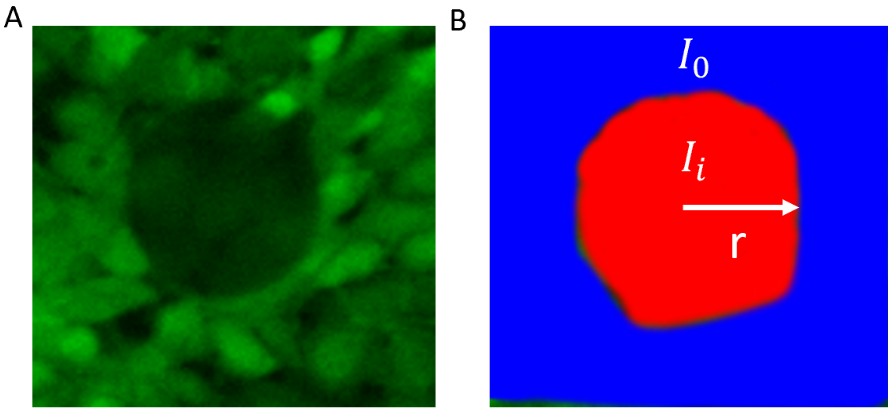

Figure S2. Demonstration of the selected area we focused on for calculating diffusion coefficient in FRAP experiments. (A) the selected square region with the photobleached circular region at the center. (B) Highlighting bleached and non-bleached areas in (A) with red and blue.

$$D_{eff} = \frac{r^2}{2\Delta t} \frac{\Delta I_i(t) - \Delta I_o(t)\frac{I_i(t)}{I_o(t)}}{I_o\left(t+\frac{\Delta t}{2}\right) - I_i\left(t+\frac{\Delta t}{2}\right)} \tag{5}$$

where r is the radius of our circluar photobleaching area, $I_i(t)$ and $I_o(t)$ refers to the recorded fluorescent intensity time series data in the bleached area and non-bleached area seperately(Fig.2B). Since our recording frame rate is 30 s/frame, we set Δt equal to 60. For comparison between different experimental conditions, we use the diffusion coefficient data between 150s post-bleaching and 750s post-bleaching. This is aimed at focusing on the steady flow of the molecule dye that is not affected by the abrupt flow right after bleaching and little to no flow caused by the unhealthy condition of cells due to too many exposures to fluorescent light at the end of recording.

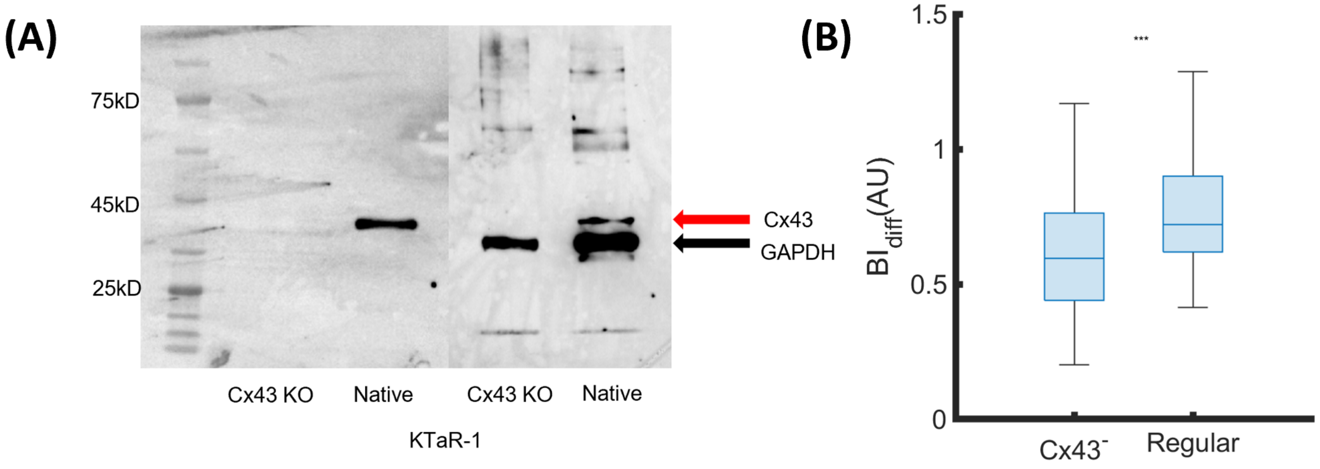

Figure S3. Western blot for Connexin 43 expression in native and CX43^{-} KTaR cells. (A) Western blot indicates absence of Cx43 band in stably-transfected CX43^{-} KTaR subcloned cells. (Left) Protein lysates (25ug) from CX43^{-} and unaltered KTaR cells probed with a Cx43 primary antibody (red arrow; 43kD). (Right) Same blot re-probed with mouse anti-GAPDH primary antibody (black arrow; 37kD). PageMark protein ladder shown (G-Biosciences). (B) Immunostaing result suggests decreased connexin 43 protein expression in CX43^{-} cells compared to regular KTaR cells. This is the boxplot to demonstrate the normalized brightness difference BI_{diff} for monolayer immunostaining experiments using connexin 43 knockout cells and regular KTaR cells. The background intensity BI_{bkg} equals 864.6 and 855.8 for the connexin 43 ko cells experiment and regular KTaR cells experiment respectively. Statistical comparisons are done with ANOVA.***P<0.001

S3. Biological characterization of native and CX43^{-} KTaR cells

a. Connexin 43 expression

We examine the connexin 43 expression in the cells using western blot (Fig. S3A). Cx43- and native KTaR cells were lysed in RIPA buffer containing protease inhibitors (Amresco, OH). The protein concentrations were determined via Pierce BCA protein assay kit and separated on a bis-tris polyacrylamide gel (Thermo Fisher, MA). Protein was transferred to nitrocellulose membrane (Bio-Rad, CA), blocked in 5% non-fat milk (LabScientific, NJ) for 1 hr, and incubated overnight in the Cx43 primary antibody (Santa Cruz Biotechnology, TX) at 4°C. The membrane was washed in TBS-T and incubated with the appropriate secondary antibody (Santa Cruz Biotechnology, TX). The membrane was then incubated with the GAPDH primary antibody (Invitrogen, MA) overnight at 4°C. The membrane was washed in TBS-T and incubated with the appropriate secondary antibody (Santa Cruz Biotechnology, TX). The proteins were imaged using SuperSignal ELISA Femto Maximum Sensitivity Substrate on an ImageQuant LAS 4000. The images were analyzed using ImageQuant TL software.

We also examine the connexin 43 expression in the cells using immunofluorescence imaging after exposing cells with periodic ATP stimuli at period of 200 seconds (Fig. S3B). For each experiment, we extract the cell non-occupied brightness intensity BI_{empty} and cell baseline brightness intensity $BI_{baseline}$ using the same methods as we previously mentioned in section S2. The background intensity will be calculated as the average of all cell non-occupied brightness intensity $BI_{bkg} = < BI_{empty} >$.

Then, we calculated the normalized brightness difference BI_{diff} between each cell baseline brightness intensity and the background intensity:

$$BI_{diff} = \frac{BI_{baseline} - BI_{bkg}}{BI_{bkg}} \tag{6}$$

This normalized brightness difference is used to evaluate connexin 43 expression.

b. Absence of synaptic communication within KTaR micropatterned biological neuron networks

Using F-actin immunofluorescent imaging, we find no visual evidence of synapses forming in the micropatterned biological neuron networks consists of KTaR cells (Fig. S4). Similarly, we have confirmed that there is no distant (iec. non-contact) electrical connection within the monolayer using a high density electrode array stimulation and electrophysiology recording (data not shown).

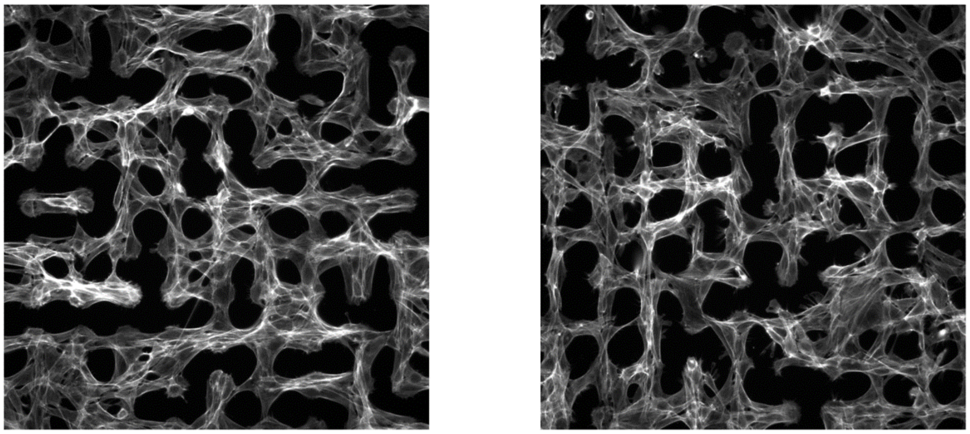

Figure S4. Immunostaining of the F-actin within KTaR MBNNs on 0.75 edge probability pattern doesn't exhibit elongated fiber structure. This indicates that KTaR cells don't have synaptic connections with each other

c. Absence of spontaneous activities in KTaR micropatterned biological neuron networks

We find KTaR monolayers exhibit little spontaneous calcium activities without ATP stimulation (Fig. S5). The results confirm the calcium dynamics reported in this study are driven by ATP stimuli.

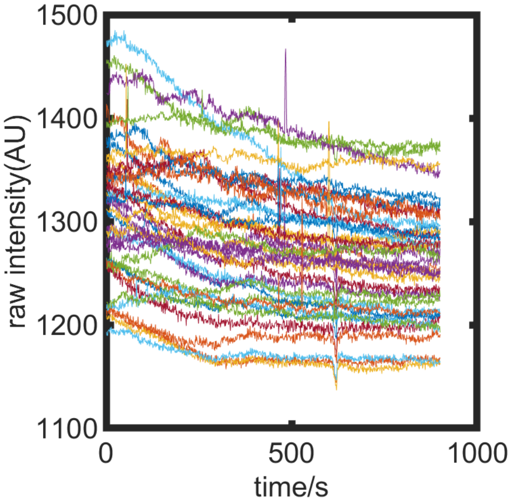

Figure S5. Raw intensity for KTaR neuron monolayer without stimulation. The graph plots the intensity for 40 cells from two repeated experiments where pure growth medium without ATP flows by the cells.

S4. Additional experimental results

Calcium dynamics is necessary for enhanced gap junction expression

We show that the enhanced gap junction formation in the micropatterned biological neuron networks require unblocked period calcium dynamics at long period (T=200 sec). As shown in Fig. S6, the enhanced gap junctions are absent in (i) periodic stimulation at short period (T=40 sec), (ii) control experiments without ATP (but with the same pattern of flow), and (iii) periodic stimulation at long period (T=200 sec) but store-operated calcium dynamics is abolished by treatment of Thapsigargin.

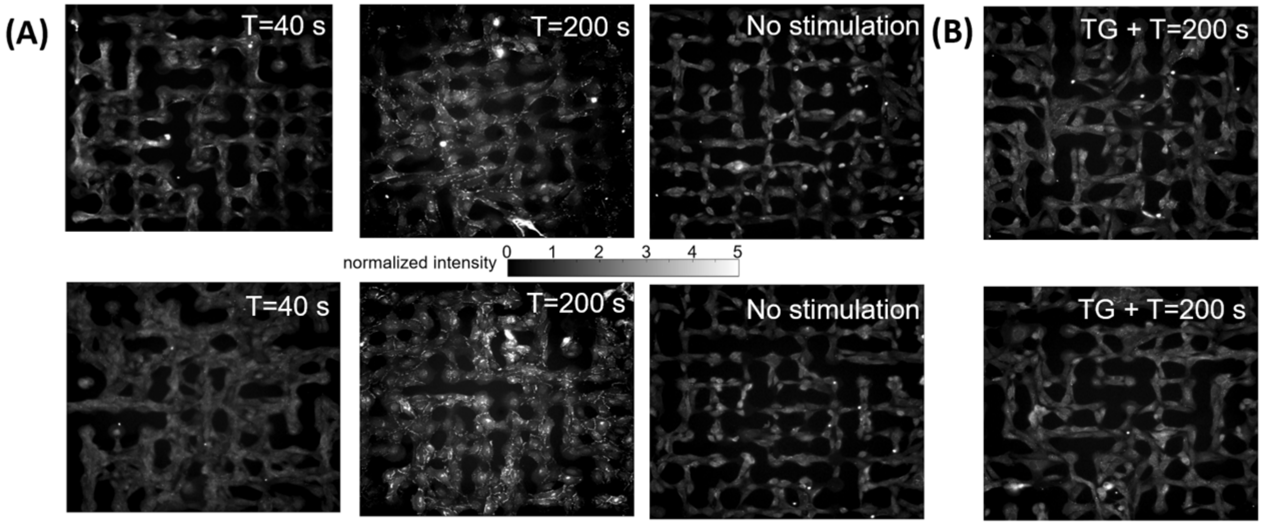

Figure S6. Side-by-side comparison of the immunofluorescent images of connexin 43 proteins of KTaR cells forming micropatterned biological neuron networks (MBNNs) under different periodic ATP stimulation with or without thapsigargin (TG) treatment. (A) The MBNNs are exposed to periodic ATP stimuli of period T = 40 seconds (left) or periodic ATP stimuli of period T = 200 seconds (middle) or no ATP stimuli but only growth medium flow (right) for 15 minutes before fixation and immunostaining. (B) The MBNNs are exposed to T = 200 seconds of periodic ATP stimulation with Thapsigargin treatment for 15 minutes. The image intensity has been normalized following the description in S2c.

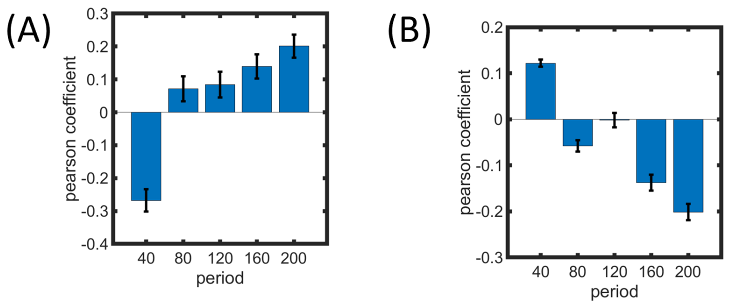

Figure S7. Linear trend analysis consistently show that connectivity of MBNNs facilitates the network synchronization for rapidly varying stimuli but destroys synchronization for slowly varying stimuli. (A) Pearson coefficient quantifying the linear trend between deviation score and edge probability (p =0, 0.5 and 0.75). Mean and standard deviation from all nodes in the field of view is shown as the bars and error bars. (B) Pearson coefficient quantifying the linear trend between node-to-node cross-correlation and edge probability (p =0, 0.5 and 0.75). Mean and standard deviation from all nodes in the field of view is shown as the bars and error bars.

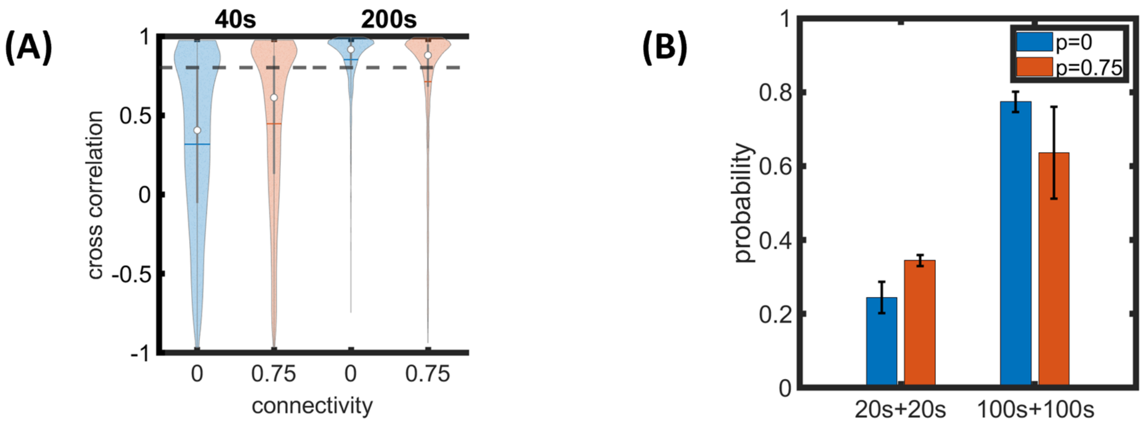

Figure S8. In the main text we present the cross-correlation analysis between neighboring nodes (Fig. 2D). Here we show consist results by analyzing the cross-correlation between individual nodes and the population, under various connectivity and period conditions. (A) Violin plots show the distribution of cross-correlation between individual node and population average. The dotted line in the graph represents the 0.8 threshold used to identify a node that is well-synchronized with population average. (B)The probability of a node to be well-synchronized with the population average.

References

1. Li, G. *et al.* Temporal signals drive the emergence of multicellular information networks. *Proc. Natl. Acad. Sci. U. S. A.* **119**, e2202204119 (2022).
2. Potter, G. D., Byrd, T. A., Mugler, A. & Sun, B. Communication shapes sensory response in multicellular networks. *Proc. Natl. Acad. Sci.* **113**, 10334–10339, DOI: 10.1073/pnas.1605559113 (2016).
